# Acute Appendicitis Presenting As Epigastric Pain Due to Incomplete Intestinal Malrotation

**DOI:** 10.7759/cureus.15088

**Published:** 2021-05-18

**Authors:** Ahmad LF Yasin, Ahmad Hisham Mohammad Sh'aban, Amman Yousaf, Ali Toffaha, Zeyad Tareq Jaleel

**Affiliations:** 1 Radiology, Hamad Medical Corporation, Doha, QAT; 2 Radiology, Hamad General Hospital, Doha, QAT; 3 Radiology, Services Institute of Medical Sciences, Lahore, PAK; 4 General Surgery, Hamad Medical Corporation, Doha, QAT; 5 Specialist Radiology, Hamad Medical Corporation, Doha, QAT

**Keywords:** acute appendicitis, incomplete, malrotation, abdominal pain, computed tomography

## Abstract

Acute appendicitis is one of the most frequent causes of acute abdominal pain. Clinical signs, blood tests, and imaging are important to confirm the diagnosis. The classic presentation consists of periumbilical abdominal pain that migrates to the right lower quadrant, guarding, tenderness, and rebound tenderness in the region. We present the case of a 51-year-old male who presented with a one-day history of worsening supraumbilical pain. Abdominal computerized tomography (CT) scan showed intestinal malrotation in which the cecum assumed a midline supraumbilical location with CT features of acute appendicitis. This case highlights that the site of pain in acute appendicitis can be altered depending upon the anatomical location of the appendix, and relying on the pain’s location can be misleading. We also suggest that patients with abdominal pain, which is not typical for acute appendicitis, should be investigated by abdominal CT if leukocytosis and inflammatory markers are raised.

## Introduction

Acute appendicitis is one of the most common causes of acute abdominal pain. The clinical presentation is usually abdominal pain in the right lower quadrant, tenderness, and rebound tenderness in the right iliac fossa. The location of the abdominal pain can vary if the appendix is in an unusual location. This could happen in patients with situs inversus totalis or midgut malrotation [1,2]. This atypical presentation can delay diagnosis and management, which might increase the risk of complications [3]. Intestinal malrotation is a congenital anomaly that arises due to incomplete or non-rotation of the midgut around the axis of the superior mesenteric artery during embryonic development [1,2]. Patients with intestinal malrotation can present during the first month of life as bilious vomiting or obstructive symptoms due to midgut volvulus or abnormal peritoneal bands (Ladd’s bands). However, most intestinal malrotations in adults are incidentally discovered [2].

In this article, we present a case of atypical presentation of acute appendicitis due to incomplete malrotation, which was diagnosed incidentally on the abdominal computerized tomography (CT) scan. To the best of our knowledge, there are fewer than 50 reported cases in the literature of acute appendicitis in the background of incomplete midgut malrotation.

## Case presentation

A 51-year-old male with no significant past medical or surgical history presented to the emergency department with a one-day history of dull supraumbilical abdominal pain. The pain was progressively worsening, initially localized to the supraumbilical region, and then migrated and became localized to the epigastrium. The pain was associated with nausea, vomiting, and anorexia. The patient had no urinary or gastrointestinal symptoms. His initial vitals were as follows: heart rate 95 beats per minute, respiratory rate 17 breaths per minute, temperature 37.2°C, and blood pressure of 115/80 mmHg.

Abdominal examination revealed epigastric tenderness, rebound tenderness, and guarding. Laboratory tests showed a white blood cell count of 5.6 × 10^9^/L with 88% neutrophils, lactic acid level of 2.9 mmol/L, and normal serum amylase and lipase. His electrocardiogram and upright abdominal X-ray were unremarkable.

**Figure 1:**
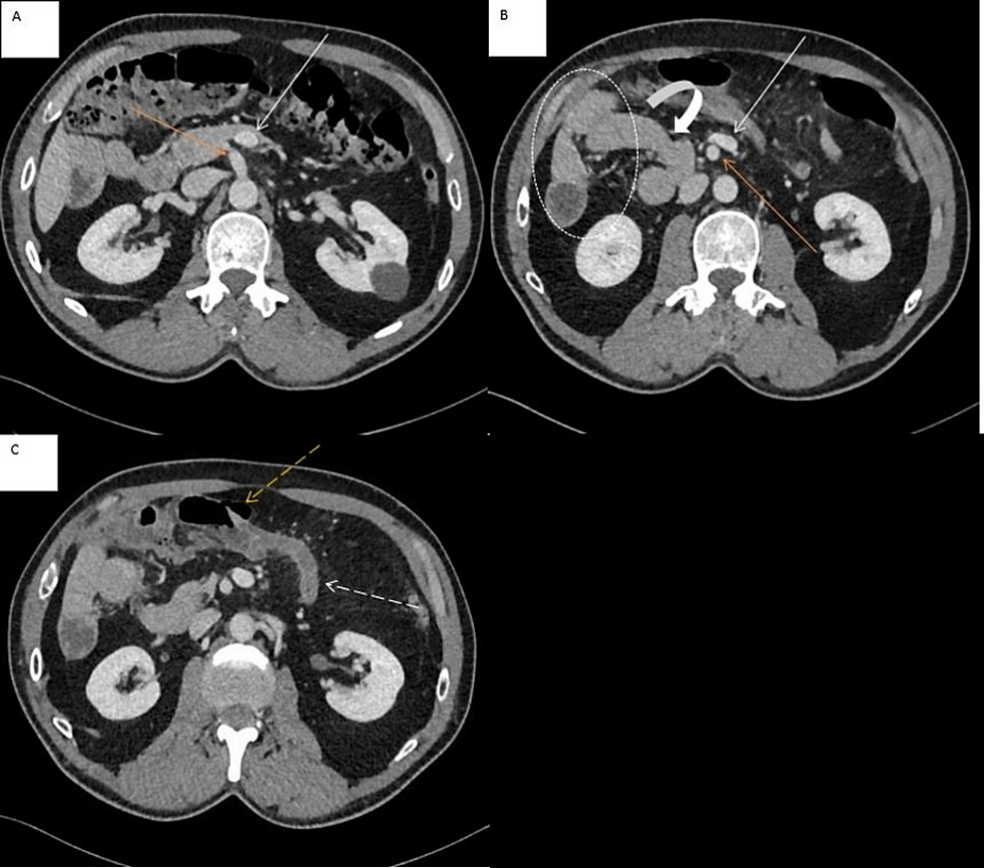
Multiple sections of axial contrast-enhanced CT abdomen. Axial contrast-enhanced CT abdomen showing SMA (orange arrow in A & B) to the right of SMV (white arrow in A & B), duodenojejunal junction to the right of the midline (curved arrow in B), small bowel jejunal loops on the right (dashed circle in B), and the cecum in the midline supraumbilical position (dashed orange arrow in C) with the arising inflamed appendix (dashed white arrow in C). CT: computed tomography; SMA: superior mesenteric artery; SMV: superior mesenteric vein

**Figure 2:**
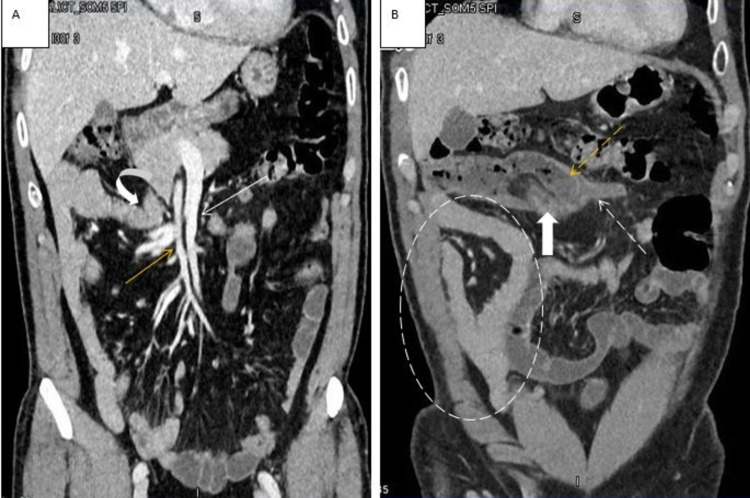
Coronal reformation of contrast-enhanced CT abdomen. Contrast-enhanced CT abdomen showing SMA (orange arrow in A) to the right of SMV (white arrow in A), duodenojejunal junction to the right of the midline (curved arrow in A), small bowel jejunal loops on the right side (dashed circle in B), and the cecum in the midline supraumbilical position (dashed orange arrow in B) with the arising inflamed appendix (dashed white arrow in B). The ileocecal valve with the surrounding fat is also demonstrated (block arrow in B). CT: computed tomography; SMA: superior mesenteric artery; SMV: superior mesenteric vein

As per the recommendation of the surgical team, an abdominal CT angiogram was performed to rule out mesenteric ischemia, which did not reveal any abnormality of the aorta and mesenteric vessels; however, incomplete intestinal malrotation was noted as an incidental finding with the inversed relationship between superior mesenteric artery (SMA) and superior mesenteric vein (SMV). Moreover, there was a right-sided duodenojejunal junction, and the major bulk of the small intestine was on the right side of the abdomen. It also showed that the cecum was located in the midline in the supraumbilical region. The appendix was arising from it, demonstrating thickened enhancing and peri-appendiceal inflammatory changes (Figures 1, 2).

Given these radiological findings, the diagnosis of incomplete intestinal malrotation with acute appendicitis was established. The patient underwent laparoscopic appendectomy with the ports inserted in the supraumbilical and right upper abdominal quadrant, and the histopathology confirmed the diagnosis of acute appendicitis. The patient had an uneventful recovery and was discharged home with no complaints on follow-up and to date.

## Discussion

Patients with acute appendicitis commonly present with periumbilical abdominal pain, which usually migrates to the right lower quadrant. Fever, nausea, vomiting, and anorexia are the other accompanying clinical symptoms. On physical examination, the patients usually have a right lower abdominal tenderness, rebound tenderness, rigidity, guarding, and positive psoas sign [4]. The typical CT findings in patients with acute appendicitis are inflamed appendix with thickened wall, not opacifying/filling with orally administered contrast, and a diameter of more than 6 mm [5]. A radiodense appendicolith is also seen in some cases.

This typical presentation can be seen in around 50% of the patients. There is a high possibility of missed or delayed diagnosis when a patient comes with an atypical presentation of acute appendicitis secondary to the unusual location of the appendix. One of the causes of the unusual location of the appendix is intestinal malrotation. Intestinal malrotation is a developmental anomaly involving the rotation and fixation of the midgut. It is rare in adults (0.1-0.5%) and usually remains asymptomatic [6]. The differential diagnosis of epigastric pain includes but is not limited to gastritis, peptic ulcer disease, gastroesophageal reflux disease, acute cholecystitis, cholangitis, pancreatitis, acute mesenteric ischemia, SMA syndrome, and acute inferior myocardial infarction [7]. In our case, we initially suspected acute mesenteric ischemia due to elevation of the lactic acid level. The intestinal malrotation and acute appendicitis were incidentally identified on the abdominal CT scan.

Intestinal malrotation happens if there is either non-rotation or incomplete rotation of the fetal intestinal loops around the axis of the SMA during the first 10-12 weeks of fetal life. It is a rare anomaly with an incidence rate of 1/500 live births [6]. In about 93% of cases in neonates, intestinal malrotation presents with bilious vomiting during the first month of life. However, it is usually asymptomatic in adults. If symptomatic, adult patients experience chronic abdominal pain in about 87% of cases [8]. The most common findings of intestinal malrotation are right-sided positioned duodenojejunal junction, right-sided location of the small bowel and left-sided location of the colon, and inverted SMA/SMV relationship [9]. These findings were noted in our patient as well.

It is crucial to differentiate between intestinal malrotation and situs inversus totalis, in which every organ is located in a mirror position to situs solitus [10]. Chest X-ray is crucial in making this differentiation. More than two-thirds of the left-sided appendicitis is due to situs inversus totalis instead of intestinal malrotation [7]. Acute appendicitis is treated by surgical removal with either an open or laparoscopic approach. The CT findings regarding the type of malrotation and the cecum location help the surgeon determine the best approach accordingly [11,12].

## Conclusions

The typical clinical presentation of acute appendicitis is altered in the presence of underlying intestinal malrotation, leading to a delay in diagnosis and management. In these situations, CT scan is a crucial problem-solving modality that can provide a prompt diagnosis of both entities. Moreover, CT scan can guide the surgeon with the best possible surgical approach. A low threshold to request an abdominal CT scan should be considered in patients with abdominal pain characteristic of appendicitis in an atypical location, especially if leukocytosis and high inflammatory markers are noted.
